# Risk factors of allergic rhinitis and its prevention strategies

**DOI:** 10.3389/falgy.2024.1509552

**Published:** 2024-11-27

**Authors:** Ruzhi Chen, Wei An, Xueting Liu, Jie Yan, Yuyi Huang, Junyan Zhang

**Affiliations:** ^1^ Guangdong Provincial Key Laboratory of Allergy & Clinical Immunology, Department of Allergy, The Second Affiliated Hospital of Guangzhou Medical University, Guangzhou, China; ^2^ Department of Nuclear Medicine, The Third Affiliated Hospital of Guangzhou Medical University, Guangzhou, China

**Keywords:** allergic rhinitis, risk factor, prevention strategy, hereditary factors, maternal situation, health status

## Abstract

Allergic rhinitis (AR) is a global disease with high prevalence. It reduces the patient's quality of life seriously. The health care and management of AR was also a heavy social burden. Specific immunotherapy (SIT) is the only curative treatment for AR that may alter the natural course of this disease. However, acceptance and compliance of SIT in AR patients are still not high and many patients are not effectively controlled. Disease prevention based on known risk factors is much more cost-effective compared to post-diagnosis treatment. There have been some reports on the risk factors of AR up to now, but the information is fragmented. This review systemically clarified the risk factors of AR including hereditary factors and family history, maternal situation & mode of delivery and feeding, personal characteristics, nutrition and food intake, personal behavior and habits, acquired environmental and chemical exposure, diseases and health status. The preventive strategies were also proposed briefly. This review was hopeful to improve people's awareness of the risk factors of AR and put forward AR prevention.

## 1 Introduction

Allergic rhinitis (AR) is a global disease characterized by symptoms of sneezing, rhinorrhea, nasal obstruction, and pruritus (1, 2). It affects about 5%–50% population in the world (3, 4). AR is associated with the onset of rhinosinusitis and asthma. They are all united airways disease and shared some similar mechanism according to the concept that upper and lower airways form a single organ (5). AR seriously reduces the quality of life of AR patients (6, 7). In addition, the health care and management of AR cause a large economic burden to the society (8).

**Figure 1:**
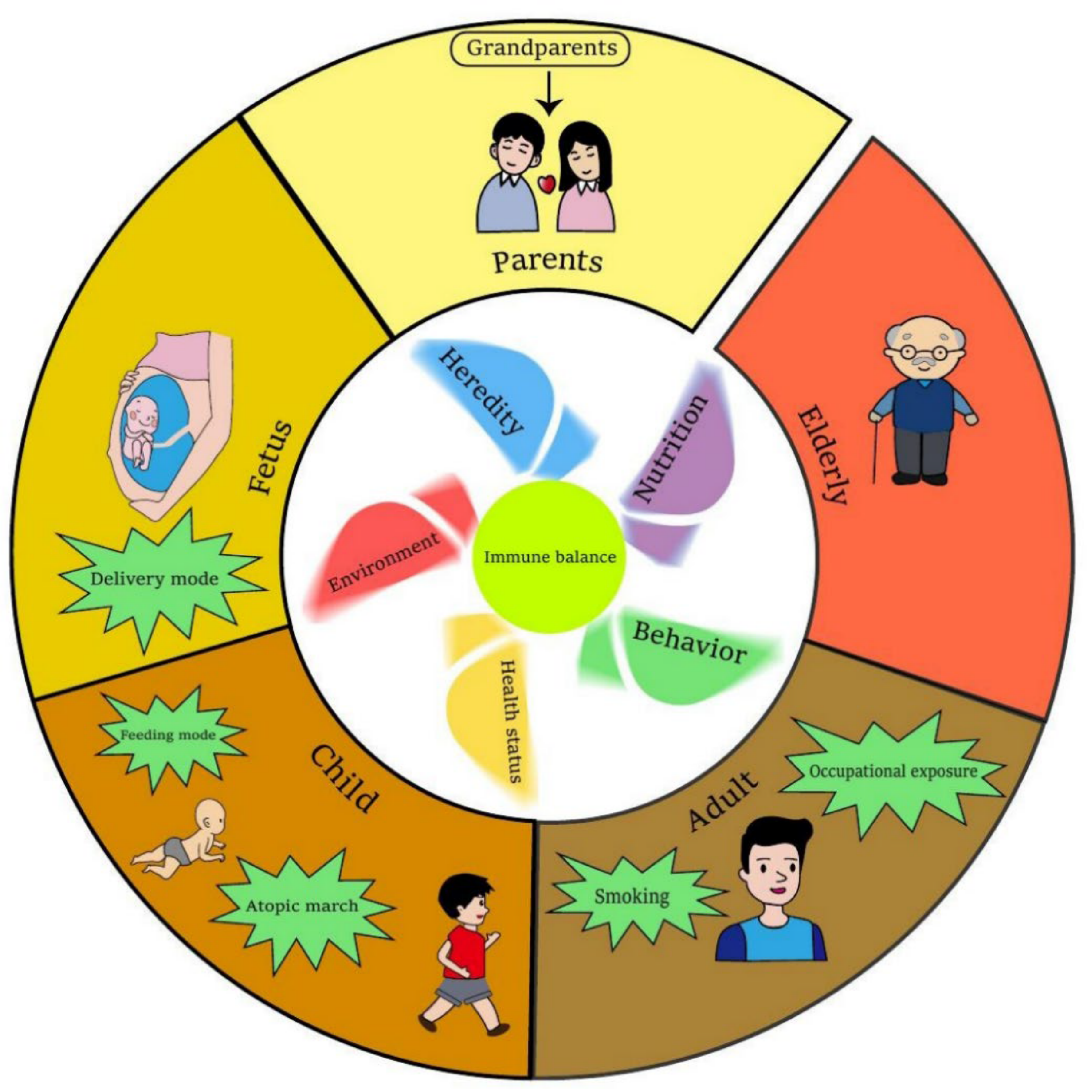
Risk of AR in humans’ whole life. The windmill in the inner ring has five blades which stand for heredity, environment, behavior, nutrition and health status of an individual. All of these aspects will influence the immune balance of human. Abnormal of these five blades contribute more or less to the immune imbalance, which induce AR development. The outer ring stands the different stages of a human's whole life. Main risk factors of AR at certain stage are highlighted. This figure was created with Adobe Illustrator.

AR is known to be a disease mediated by Th2 type immune response. Genetic factors and environmental factors are both involved in the occurrence and development of AR, but its specific pathogenesis still needs further research (9). Pharmacology and specific immunotherapy can effectively alleviate the symptoms of AR and slow down the process of AR, but there are still many AR patients who are not under good control with low and bad compliance for the treatment in the real world (9, 10). Compared to treatment after diagnosis, prevention of AR based on the potential risk factors should be much more cost-effective and valuable. However, practice on AR prevention is still blank now. So far as we know, there have been some reports on the risk factors of AR. The information is fragmented, and readers couldn't clearly and systematically understand. This review clarified the risk factors of AR through the human's whole life (Figure 1). The important risk factors of AR with larger consensus are discussed, while some ambiguous viewpoints are not involved. The preventive strategies based on these risk factors were also proposed briefly. We hope this narrative review be helpful to improve people's awareness of the risk factors of AR and put prevention strategies to move forward.

## 2 Hereditary factors/family history of allergies

Hereditary factors play an important role in allergic diseases including AR. Parents with allergic diseases will highly increase the risk of allergic rhinitis in their offspring (11). Large-scale genome-wide association studies (GWAS) indicated that the single nucleotide polymorphisms of many human genes are associated with AR (12, 13). As shown in Table 1, these genes are divided into several categories by their feature and functions. There are pathogen-associated molecular patterns: such as TLR1, TLR4, TLR6, TLR7, TLR8, TLR10, and CD14; alarmins such as IL-33 and TSLP; chemotactic and adhesion molecules including CXCR5, and VCAM-1; cytokines and their receptors such as IL-1, IL-4, IL-6, IL-13, IL-17a, TNF-α, TGF-β, IL1R, IL7R, IL-9R, IL-18R1; immunoglobulin receptors including FCRL3, FCER1B and FCER1G; co-stimulatory molecules and immunomodulators such as CTLA-4, TNFSF4, CD207; epithelial barrier function related genes such as filaggrin and GJA10; genes related to DNA repair capacity and maintaining genome integrity such as hOGG1, XRCC1and LIG3. Other genes such as ACE, SP-A, PTPN22, ADAM33, etc. are also involved.

**Table 1:** The heredity factors of AR.

Category/subcategory		Hereditary factors associated with AR
Genes	HLA genotypes	HLA-B, HLA-DQB1, HLA-DPB1;
	Pathogen-associated molecular patterns	TLR1, TLR4, TLR6, TLR7, TLR8, TLR10, CD14;
	Alarmins	IL-33, TSLP;
	Chemotactic and adhesion molecules	CXCR5, VCAM-1;
	Cytokines and receptors	IL-1, IL-4, IL-6, IL-13, IL-17a, TNF-α, TGF-β, IL1R, IL7R, IL-9R, IL-18R1;
	Immunoglobulins and their receptors	FCRL3, FCER1B, FCER1G;
	Co-stimulatory molecules/immunomodulators	CTLA-4, TNFSF4, CD207;
	Epithelial barrier function	Filaggrin, GJA10;
	DNA repair capacity/maintaining genome integrity	hOGG1, XRCC1, LIG3;
	Other genes	ACE, SP-A, PTPN22, ADAM33, BLK, VDR, PLCL1, MRPL4, WDR36, ENTPD6, CAMK4, CAPSL, DDX6, AKR1E2, TMEM232, FERD3l, DLG1, LRRC32, BCAP, CBLN1, CDK2AP1, MTRFR;
Transcription factors	JAK1, BACH2, RORA, EMSY;	
LncRNAs	NEAT1, Lnc-GAS5, ANRIL, LncRNA, FAM239A;	
MicroRNAs	let-7, miR-224, miR-18a, miR-135a, miR-206, miR-338-3p, miR-498, miR-155, miR-126, miR-205, miR-375, miR-19a, miR-26a, miR-16;	
Epigenetics	Methylation, histone acetylation, Ubiquitination, Phosphorylation;	

These genes mentioned above are involved in cell migration, chemotaxis and adhesion, maturation and activation of immune cells, antigen presentation, pathogen recognition and activation of innate immunity, pro-inflammatory effects, T helper cell function and regulation, immunoregulation, etc. Transcription factors or transcription repressors such as JAK1, BACH2, RORA, and EMSY are also reported in risk of AR, as well as some lncRNAs and microRNAs listed in Table 1. Gene-environment interaction effects and gene epigenetic modification such as methylation, histone acetylation, ubiquitination, and phosphorylation are reported strongly associated with AR (14). Furthermore, polygene rather than a single gene contributed to the occurrence and development of AR. Shared genetic mechanisms for AR and other allergic diseases such as asthma and atopic dermatitis also existed (1). And only a few genes related to disease-specific effects in AR. Although hereditary factors are critical risk factors for AR; we could not do too much for that except for reducing the negative impact of gene-environment interaction and recognizing the necessity of AR prevention.

## 3 Maternal situation & mode of delivery and feeding

Pregnancy is a critical time window for the early life programming period of the fetal immune system (15). Maternal situations such as food and nutrition intake, behaviors, health status, and living environment as well as the mode of delivery and feeding are all associated with the risk of AR in their offspring (as shown in Table 2).

**Table 2:** Risk factors of AR and related mechanism.

Classification	Certain risk factors of AR (Decrease ↓/Increase ↑)		Related mechanism	Refs.
Hereditary factors and family history	Genes and family history	Susceptibility genes ↑ Family history ↑	Gene expression and epigenetic regulation	
				(11, 12)

Maternal situation & mode of delivery and feeding	Nutrition and food intake	Healthy diet ↓ Unhealthy diet ↑	Maternal immune balance is affected	(16)
	Maternal behaviors	Smoke exposure ↑ Short sleep duration ↑ Physical inactivity ↑ Excessive screen exposure ↑	Abnormal maternal immunity and inflammation	(17)

				(18)

		Contraceptive use ↑	Abnormal sex hormone level	(19)
		Intake of phthalates ↑	Chronic toxicity and adjuvant effects	(20)
	Living environment	Air pollution ↑ Living on a farm ↓	Substances go across the placenta and affect fetal immunity	(21)
				(22)
	Health status	Anxiety ↑ Depression ↑	Endocrine disorders and inflammatory activation	(23)
		Gestational diabetes mellitus ↑ Obesity ↑	Endocrine disorders and immune imbalance	(24, 25)
	Mode of delivery	Cesarean section ↑ Vaginal delivery ↓	Neonatal gut microbiota colonization	(26–29)
	Mode of feeding	Breastfeeding ↓ Non-breastfeeding ↑	Beneficial ingredients in breast milk	(30, 31)
Personal characteristics	Gender & Age	Prepubertal male ↑ Postpubertal female ↑	Dynamic changes of sex hormones	(32–35)
		Elderly ↓	Decreased immunity, lower IgE	(36)
	Blood type	Type O blood ↑	Diverse glycoconjugate expression profile and content	(37)
	BMI/Obesity	Obesity ↑	Abnormal endocrine hormones such as leptin affect immunity	(38–41)
Nutrition and food intake	Vitamin intake	Vitamin D ↓ Vitamin C ↓ Vitamin E ↓	Regulation of inflammation and immune response	(42–45)
	Dietary fiber	Supplement ↓	Th2 responses inhibition Maintain gut flora homeostasis	(46, 47)
	Trace elements	Se ↓, Zn ↓ Cu ↑, Ni ↑, Cd ↑, Pb ↑	Regulation of immune balance and inflammation	(45, 48, 49)
	Probiotic	Supplementation ↓	Maintain gut flora homeostasis	(50, 51)
	Fat intake	Saturated fat ↑ PUFAs ↓	Inflammatory activation and regulation	(52–54)
Personal behavior and habits	Smoking	Smoking ↑ Second-hand smoke ↑	Airway irritation and inflammatory activation	(55, 56)
	Physical activity	Moderate exercise ↓ Intense exercise ↑ Sedentary conditions ↑	Regulation of oxidative stress and immune response	(57–59)
	Life rhythm	Irregular life with disruptions in the circadian clock ↑	Abnormal changes in the metabolic rhythm and hormone levels affect immunity	(60, 61)
Acquired environmental and chemical exposure	Living environment	Dampness and mold ↑ Dense traffic ↑ Farm environment ↑ Ambient temperature variety ↑ Extreme heat events ↑	High allergen exposure and inflammatory activation	(62–67)
	High-risk occupations	Hairdresser ↑ Baker ↑ Animal contact ↑ Cleaning worker ↑ Farmer ↑ Pulp mill worker ↑ Automobile repair worker ↑	Occupationally exposure to allergens and chemicals frequently	(68–75)
	Chemical exposure	Cleaning products ↑ Pesticides ↑ Phthalates ↑ Antibiotics ↑ Antacids ↑ Acetaminophen ↑	Irritation to the respiratory tract, adjuvants effect, metabolic disorders and immune imbalance	(72, 73, 76–82)
Diseases and health status	Psychiatric diseases Immune diseases Microbial pathogens infection	Increase ↑ Decrease ↓	Diseases affect immune balance	

PUFAs, polyunsaturated fatty acids.

A healthy maternal diet during pregnancy can reduce the risk of allergic rhinitis in offspring, otherwise, the opposite is also true (16). Healthy food includes a variety of seafood represented by fish, fresh fruits and vegetables, and a variety of dairy products. Beneficial components in food include vitamin D, n-3 polyunsaturated fatty acids, probiotics, etc. Unhealthy foods include a variety of improperly cooked foods or diets high in meat, carbonated drinks, and so on. Harmful components of food include heterocyclic amines, polycyclic aromatic hydrocarbons, high concentrations of sugar, etc. More detailed information was illustrated together in the section on food and nutrition intake.

Environmental tobacco smoke exposure during pregnancy may affect the occurrence and development of allergic diseases in children through epigenetic mechanisms, including histone acetylation, microRNA expression, and DNA methylation (83). The harmful components nicotine, which up-regulates a variety of inflammatory factors including IL-8 can cross the placenta and affect the development of the fetal immune system (17). Maternal short sleep duration, physical inactivity, and excessive screen exposure during pregnancy were also associated with an increased risk of AR in offspring (18). The use of oral contraceptives before pregnancy may lead to epigenetic changes, and these changes may be transmitted to the embryo, leading to the occurrence of allergic diseases such as rhinitis in childhood. Furthermore, taking contraceptives lead to abnormal secretion of progesterone and other hormones in the mother's body, which may not only affect the development of fetal immunity but also alter the composition of the vaginal microbiome (19). Other harmful substances such as phthalates and their metabolites, cadmium(Cd), and plumbum(Pb) in maternal blood also have certain effects on the pathogenesis of allergic rhinitis in offspring (20, 48).

Maternal exposure to air pollutants such as PM2.5, mold, volatiles from newly refurbished furniture, and toxic organic solvents during pregnancy may increase the risk of rhinitis in offspring (21, 84). Studies have shown that prenatal exposure to air pollutants leads to maternal Th1/Th2 imbalance through the induction of IL-25 and IL-13 (21, 85). However, prenatal exposure to a farm environment is protective, which can be explained partly by high exposure to microorganisms, particularly bacterial endotoxin in the farm environment, which is helpful to prime the development of fetal immunity (22).

Maternal anxiety and depression during pregnancy, gestational diabetes mellitus (GDM), as well as obesity, can increase the risk of allergic diseases in offspring (23–25). Depression and anxiety are often accompanied by persistent hypersecretion of cortisol (23). Mothers with GDM exhibit elevated levels of TNF-a, leptin, and visfatin, while adiponectin was decreased (24). Maternal obesity during pregnancy is always accompanied by insufficient intake of vitamin D and an imbalance of intestinal flora, which can increase the risk of AR (25).

The mode of delivery was reported to affect the prevalence of AR in the offspring. The cesarean section increases the risk, while vaginal delivery is the opposite (26–29). Children delivered by cesarean section had no opportunity to be inoculated vaginally with beneficial bacteria from their mothers at birth, such as bifidogenic microflora, which makes them hard to build a balance of gut microbiota in early life (29). The imbalance of gut microbiota impairs the natural development of the immune system and increases the risk of allergic disease in the future.

Breastfeeding reduces the risk of AR in offspring after birth (30). Relevant mechanisms were systematically introduced as follows: 1. Breastfeeding promotes the maturation of the gastrointestinal mucosa. 2. Breastfeeding can reduce the occurrence of infectious diseases in infants and regulate their intestinal flora, thereby reducing the possibility of sensitization. These benefit from the secreted antibodies in the breast milk. 3. Human milk contains functional immunomodulatory and anti-inflammatory factors (31).

## 4 Personal characteristics

Personal situations such as age, gender, ABO blood group, and obesity are all related to the risk of AR.

The prevalence of AR in the population increases steadily with age in the early life period, reaches the peak at about 20 years old, and then gradually decreases, reaching the lowest at 65–85 years old. This trend happened both in male and female (86). IgE against specific allergens decreases with age in elderly AR patients (36). Rhinorrhea and nasal congestion are the most common symptoms in elderly and adult AR patients, respectively. Compared to adult patients, elderly patients had a lower response to 4 weeks of AR treatment recommended by existing guidelines (87).

There is a “gender shift” in AR prevalence: from a male predominance before puberty and finally to a female predominance after puberty, in which sex hormone levels may play a key role (32, 33). Studies have shown that androgen can reduce allergic airway inflammation by increasing the T-reg/Th2 ratio in the lung (34). On the contrary, estrogen increases the risk of AR by mechanisms such as promoting the polarization of Th2, priming B cells with the potential for IgE expression, and increasing the degranulation of mast cells and basophils (35). The dynamic changes of sex hormones make the “gender shift” in AR prevalence. ABO blood groups were also found to be associated with allergic diseases. Blood group O had greater susceptibility to AR and asthma, while non-O blood groups were related to AD (88, 89). This is possibly related to the diverse glycoconjugate expression profile and content induced by the ABO gene (37).

The risk of AR is significantly increased in obese people, which may be related to the endocrine function of adipose tissue and the change in immune response in obese people (38, 39). Studies indicated that leptin secreted by adipose tissue in obese AR patients reduced immune tolerance to allergens by induction of proinflammatory cytokines, such as IL-6 and TNF-a, enhancement of Th2 and Th17 inflammation, and inhibition of T-reg cell function (90, 41).

## 5 Nutrition and food intake

Adequate vitamin supplementation is helpful for AR. Specifically, vitamin D mitigates allergic inflammation through augmentation of IL-10 production, suppression of TNF, IL-2, and IL-5, and inhibition of excessive macrophage activation (42, 43). Vitamin C functions as an important antioxidant to diminish airway inflammation (44). Vitamin E may alleviate AR symptoms by managing IL-4, IL-5, and other Th2-type cytokines, serum IgE, and histamine levels (45).

Dietary fiber demonstrates a protective role for AR (46). A plant-based, high-fiber regimen notably invigorated favorable gut bacteria, reducing circulation levels of systemic inflammatory markers including IL-6 (47). The intake of trace elements is also associated with AR. Lower serum concentrations of selenium (Se) and zinc (Zn) were found in AR patients, while copper (Cu) and nickel (Ni) were increased on the contrary (49, 91). Selenium triggers the human body's expression of glutathione peroxidase, further participating in inflammation regulation and affecting the Th1/Th2 balance (45). Zinc supplementation diminished serum IgE levels and downregulated IL-6 and TNF-α secretion in mice (92). Cadmium influences immune cell subset activation and IgE production, while blood plumbum positively correlates with eosinophil IgE levels and T cell dysregulation (48).

Probiotics supplementation was reported to lessen AR risk, which can help to keep the balance of intestinal flora and inhibit inflammation by immune protection and T-reg regulation (50, 51). A high abundance of Prevotella may have a protective effect on the development of AR (93). Lactis BB12 and E. faecium L3 supplementation mitigate AR symptoms indicating the benefit of probiotics for allergic diseases (94).

High-fat foods have demonstrated an enhancement of neutrophilic airway inflammation (52). While polyunsaturated fatty acids (PUFAs) such as eicosapentaenoic acid (EPA), docosahexaenoic acid (DHA), and arachidonic acid have protective effects on AR (53, 54). Fast food, which typically includes lots of saturated fatty acids, trans fatty acids, carbohydrates, and preservatives, is linked to an augmented risk of AR by potentially influencing the body's inflammatory and immune responses (95, 96). In addition, unhealthy dietary habits such as snacking between meals and eating dinner less than 1 h before bedtime both increased the risk of AR (96).

## 6 Personal behavior and habits

Individual behavior, habits, and lifestyle patterns such as smoking, physical activity, and life rhythm can affect the development of allergic rhinitis.

Smoking and exposure to second-hand smoke are risk factors for AR, and the types of cigarettes are not limited to traditional cigarettes but also include heated tobacco products, electronic cigarettes, and so on (55, 56). Tobacco smoke contains many toxic chemicals and carcinogens such as acrolein, acetaldehyde, and formaldehyde, which directly irritate the respiratory tract, and may also affect inflammatory processes in human and animal studies. It leads to increased eosinophil and affects the recruitment of inflammatory cells through the secretion of cytokines and chemokines, leading to airway remodeling (56). It has also been shown that cigarette smoke exposure leads to a significant increase in nasal levels of various cytokines such as IL-4, IL-5, and IL-13, which can lead to Th2 polarization (56, 97).

Physical activity correlates with the risk of AR. Moderate exercise mitigates, whilst intense exercise and sedentary conditions precipitate the opposite effect (57–59). Moderate exercise attenuates the oxidative stress response and regulates the immune cell expression profile, including reducing IL-4, IL-5, and IgE antibody production, consequently lessening AR severity and diminishing AR incidence (98). Intense exercise elevates the probability of allergic rhinitis. Intense exercise heightens Th2 cytokine production and elevates the count of nasal mucosal neutrophils, leading to the stimulation of T cells and promoting eosinophil aggregation (58).

Disturbances or disruptions in circadian clock activity will enhance the susceptibility and severity of allergic diseases (60, 61). Any disruption in the biological clock results in alterations in the metabolic rhythm and hormone levels such as glucocorticoids, affecting the metabolism, expression, and distribution of several immune cells like mast cells and eosinophils. These alterations in immune cell behavior heighten allergic risks.

## 7 Acquired environmental and chemical exposure

Living and occupational environment as well as chemical exposure are closely related to the risk of AR. High humidity that benefits mold growth, an environment with severe air pollution or with a high concentration of allergens, ambient temperature changes, and extremely hot weather are all the risks of AR (62–67). However, household use of temperature and humidity control machines and air purifiers all have a protective effect (99, 100).

High humidity environment fosters mold growth. Specifically, Curvularia lunata, Aspergillus spp., Streptomyces, and Aspergillus versicolor in the atmosphere can augment the probability of AR (101). Microbial volatile organic compounds (MVOCs) such as extracellular polysaccharides, and mycotoxins in the fungal cell wall architecture may instigate detrimental immune responses in humans. Mold exposure may lead to repeated irritation of the respiratory tract and immune activation, thereby leading to long-term inflammatory responses (62).

Air contaminants such as sulfur dioxide, PM2.5, assorted dust, and diesel exhaust particulates are exceedingly concentrated in the atmosphere adjacent to traffic thoroughfares with intense traffic volumes. These airborne pollutants not only induce direct harm and irritation to the respiratory apparatus but also impact the body's oxidative stress and inflammatory reaction. Air pollutants yield reactive oxygen species (ROS) and free radicals damage airway epithelial cells leading to airway inflammation and airway hyperresponsiveness. Inhalation of air pollutants may also affect the function of macrophages, neutrophils, T cells, and other immune cells in the body, affecting the production and secretion of a variety of cytokines, adhesion factors, and proteases (64, 102, 103).

Living on a farm and maintaining domesticated animals are vulnerability factors for AR, which may be related to increased exposure to endotoxin, pollen, animal dander, and other allergens (104, 105). However, this pattern can be reversed during one's early life. According to the hygiene hypothesis, preschool exposure to these substances in a “dirty” environment can facilitate the development and maturation of children's immune systems, which protect them from future allergies and sensitivity (106). On the contrary, deficient exposure could escalate the susceptibility to allergic diseases (107).

Environmental temperature changes and extreme heat events are associated with increased AR. Researchers propose that extreme heat events potentially increase human exposure to high concentrations of pollen allergens by affecting the plant flowering period (66, 67).

Some special occupational personnel, such as hairdressers, bakers, animal breeders who frequently contact with animals, cleaning workers, farmers, pulp mill workers, and automobile repair workers have a high incidence of AR (68–75). Occupational exposure to various prevalent allergens, including dust mites, dust, dander, and pollen will increase the risk of sensitivity, which can explain the higher risk of AR among workers engaged in animal management as well as bakers (2). Hairdressers and auto repair workers have a high frequency of exposure to chemical reagents such as perfume, cosmetics, bleach, and synthetic metalworking fluid. Cleaning workers and farmers have an increased exposure to cleaning agents and pesticides, respectively. Workers in pulp manufacturing plants are exposed to ozone more frequently, and ozone may promote the polarization of Th2 cells in the body (108).

Chemical exposure is similar to environmental exposure. The usage of cleaning products, pesticides, passive intake of phthalates, or taking some special medicine such as antibiotics, antacids, and acetaminophen are all risks of AR (72, 73, 76–82). Chemical cleaners, such as diluted bleach, not only cause strong irritation to the respiratory tract but also may act as adjuvants to affect the immune response (72, 109). Certain pesticides, especially organophosphorus pesticides, inhibit acetylcholinesterase activity, leading to the accumulation of acetylcholine, which induced the abnormal of nasal glands (77, 110). Phthalates, pervasive plasticizers in everyday life, particularly di-2-ethylhexyl phthalate (DEHP) could potentially heighten the risk of allergic reactions (111). The use of antibiotics such as cephalosporins and macrolides especially in childhood caused gut flora imbalance and maturation disorders of the immune system, which in turn facilitated the Th2 immune response and development of allergic diseases (80, 112, 113). Antacids not only cause intestinal dysbiosis but also increase sensitivity to ingested antigens by reducing protein breakdown in the stomach (79). Acetaminophen may affect cyclooxygenase-2 activity by promoting prostaglandin E2 synthesis, which in turn promotes the Th2 response (82).

## 8 Diseases and health status

### 8.1 Psychiatric diseases and AR

Research demonstrates that psychiatric diseases such as anxiety, depression, attention deficit hyperactivity disorder (ADHD), and post-traumatic stress disorder (PTSD) augment the vulnerability to AR (114–117). The paraventricular nucleus of the hypothalamus is commonly activated and the secretion of corticotropin-releasing hormone (CRH) is increased in patients with anxiety and depression. CRH stimulates the secretion of corticosteroids in a series of pathways, which in turn promotes the production of Th2 cytokines. CRH also increased epinephrine and norepinephrine production, thereby inhibiting IL-12 production (115). ADHD may be a high inflammation and immune-associated disease (118). A study found that the positive rate of skin prick tests for a variety of allergens in ADHD patients was greater than that in the control group (119). PTSD is not only involved in promoting low-grade systemic inflammation but also may lead to the methylation of immune-related genes, which increases the risk of allergy (117). Diseases and health status associated with AR are shown in Table 3.

**Table 3:** Diseases and health status associated with AR.

Classification	Certain diseases and risks for AR (Decrease ↓/Increase ↑)	Main mechanism	Refs.
Psychiatric diseases	Anxiety ↑ Depression ↑ ADHD ↑ PTSD ↑	Inflammatory activation and common genetic susceptibility	(114–117, 119)
Immune diseases	Allergic diseases: Atopic march ↑ Food allergy ↑ Atopic dermatitis↑ Autoimmune diseases: Kawasaki disease ↑ Alopecia areata ↑ Psoriasis ↑ Ankylosing spondylitis ↑	Similar hereditary factors Epithelial barrier dysfunction increases susceptibility of sensitization	(120–123)
		Abnormal immunity Immune imbalance	(124–127)
Microbial pathogens infection	Viruses:		
	Rhinovirus ↑	Nasal barrier disruption	(128, 129)
	HIV ↑ HBV ↑	Enhancing Th2 response	(130–132)
	HTLV-I ↓ Measles virus ↓	Enhancing Th1 response	(133, 134)
	Bacteria:		
	S. aureus ↑	Enhancing Th2 response	(135)
	Mycobacterium tuberculosis↓ Salmonella ↓ Helicobacter pylori ↓	Enhancing Th1 response	(136–138)
	Chlamydia:		
	Chlamydia pneumoniae ↓	Enhancing Th1 response	(139)
	Parasites:		
	Schistosoma mansoni ↓ Schistosoma japonicum ↓ Angiostrongylus cantonensis ↓ Schistosoma haematobium ↓	Suppression of Th2 response Inducing suppressive cytokines released from regulatory lymphocytes Switching IgE to IgG4	(140–143)

ADHD, attention deficit hyperactivity disorder; PTSD, post-traumatic stress disorder; HTLV-I, human T-lymphotropic virus type I.

### 8.2 Atopic march and AR

The theory of atopic march indicates that allergic diseases occur in a time order. Atopic dermatitis and food allergy in infancy would gradually develop into allergic rhinitis and allergic asthma when they grow up (120). Previous studies also certified that atopic dermatitis and eczema increased the risk of AR (121). These patients demonstrate compromised epithelial functionality, augmented epidermal sensitivity, and IgE production (122). Post-sensitization, T cells can infiltrate the respiratory tract and instigate hypersensitive reactions in the upper airway (123). Although the mechanisms underlying the atopic march are not fully clear. It still provides a perspective for the prediction and prevention of allergic diseases.

### 8.3 Autoimmune diseases and AR

Autoimmune diseases such as Kawasaki disease, alopecia areata, psoriasis, and ankylosing spondylitis are associated with the risk of AR (124–127). Kawasaki disease instigates cytokine storm, highly activating the immune system. Levels of IgE antibodies, Th2 cytokines IL-4, IL-5, and eosinophil cationic protein in patients are all elevated, thereby triggering Th2 immune-related responses *in vivo* (124, 144). Immunological dysregulations with overexpressed of both TH1 and TH2-related markers existed in patients with alopecia areata. The prevalence of AR and asthma is higher in the population with alopecia areata than in the control (125, 145). Psoriasis with high Th17 responses was also found to be associated with allergic rhinitis and asthma (126). Whereas ankylosing spondylitis augments Th2 responses, thereby escalating AR risk (127).

### 8.4 Microbial pathogens infection and AR

Virus infections were associated with AR susceptibility. One important virus is Rhinovirus. Rhinovirus (RV) infection may destroy the integrity of the nasal mucosal barrier and facilitate the allergens to penetrate the tissue (128, 129). HIV infection could foster IgE synthesis and augment the Th2 response, which increases the likelihood of developing allergic diseases (130, 131). AR prevalence also escalates in hepatitis B virus (HBV) carriers. It might be attributed to the absence of HBV surface antigen-specific Th1-type immune responses among carriers, which potentially leads to an enhanced Th2 response (132). However, infection of Human T-lymphotropic virus type I (HTLV-I) and measles virus exhibit beneficial effects against AR by enhancing the Th1 response and inhibiting the Th2 response in humans (133, 134).

Specific bacterial infection influence AR diversely. Concurrent research revealed that individuals with AR possess elevated S. aureus in their noses. S. aureus-generated enterotoxin can function as a superantigen to elevate IL-8, and IL-13 levels and diminish IFN-γ secretion that intensifies the Th2 response in humans (135). Mycobacterium tuberculosis and Salmonella, Helicobacter pylori, and Chlamydia pneumoniae infections are reported to lessen the risk of AR (139–146). Upon host infection, the Th1 response heightens to facilitate pathogen elimination, thereby reducing the Th2 response.

Parasitic infections such as Schistosoma mansoni, Schistosoma japonicum, Angiostrongylus cantonensis, and Schistosoma haematobium mostly are “protective” for allergic diseases (140, 141). Studies of the inverse association between parasitic infections and allergy indicated that parasitic infections may “protect” allergy by inducing suppressive cytokines released from regulatory lymphocytes, ablating the eosinophil response, making effector T helper 2 (Th2) cells enter anergic state and switching antibody production from IgE to the non-inflammatory isotype IgG4 (142, 143).

## 9 Prevention strategies for AR based on risk factors

Practice of AR prevention is mostly stay in the concept until now. With a deepening understanding of the pathogenesis and risk factors of AR, several corresponding prevention strategies for AR are proposed as follows. Although some strategies seem avant-garde and need further certification by evidence-based medicine.

1. AR exhibits inheritance patterns. Several susceptibility genes with AR were found by GWAS. Adenovirus-mediated gene therapy was proposed to be a promising therapy for allergy treatment. Gene therapy against these abnormal genes before AR onset based on a personal gene map provides a feasible way not only for AR treatment but also for prevention if the therapy is safe and permitted by medical ethics.
2. Pregnant parents especially mothers have a great influence on the physical condition of their offspring. Maintaining maternal health during this period was very important. Pregnant women must adopt a nutritious and balanced diet, including the consumption of more fresh fruits, vegetables, fish, and yogurt, while diminishing foods with high sugar and fat intake, which is helpful to supplement beneficial ingredients as well as to reduce the harmful ingredients. Moreover, regular exercise, ideal body weight, low stress, and a good mood during pregnancy will foster a favorable environment for the development of the fetal immune system. Avoidance of smoke or other pollutants during pregnancy is beneficial due to the detrimental impact of these substances on both inflammatory responses and fetal growth via the placenta.
3. Vaginal delivery and postnatal breastfeeding are instrumental in reducing allergic disease risks. Because balanced vaginal microbiota supports immune system development and breast milk provides nutrients and postpones allergen exposure, they offer additional protection. It is necessary to encourage mother to choose vaginal delivery and postnatal breastfeeding for their offspring.
4. Nasal recurrent infection by microbial pathogens may destroy the integrity of the nasal mucosal barrier, making humans much more susceptible to environmental allergens. In addition, some ingredients of these pathogens are potentially allergens which can cause sensitization (147). Therefore, prevention of nasal infections through vaccination against certain pathogens in early life may be benefit for AR prevention. It's worthy of further study.
5. The phenomenon of atopic march existed and the theory was reasonable. According to these, early good control of allergic diseases such as atopic dermatitis, eczema, and food allergy in infancy is helpful to prevent the possibility of allergic rhinitis and allergic asthma in one's childhood.
6. A healthy lifestyle for an individual such as a normal sleep and rest schedule, moderate physical exercise, balanced nutritional intake and healthy eating habits, a positive attitude towards life, and maintaining a good mental state are all crucial for keeping healthy. Keeping in a good environment, no smoking, less exposure to toxic and harmful substances, safety protection for some special occupational exposure, early exposure to allergens during early life (especially the immune establishment period), avoiding excessive hygiene, and maintaining the diversity and homeostasis of gut flora is helpful for people to keep away from AR and other allergic diseases.

## 10 Summary

The risk factors of AR were clarified systemically in this review. Hereditary factors and family history, maternal situation & mode of delivery and feeding, personal characteristics, nutrition and food intake, personal behavior and habits, acquired environmental and chemical exposure, diseases and health status are all involved in disease susceptibility of AR. It seems that we cannot do too much about our genes, sex, and blood group, but we can choose a healthy lifestyle, no matter whether we are pregnant parents or not. We should intake balanced nutrition and develop good living habits. We can improve our living and working environment which are very important to us. All of these can help us to keep good immunity and healthy status. These can help us to keep away from AR and other diseases to our best. We hope this review helped improve people's awareness of the risk factors of AR and put forward AR prevention steps. It's a positive response to advocacy of European Forum for Research and Education in Allergy and Airways diseases group (EUFOREA) that aim to reduce the prevalence and burden of chronic allergic and respiratory diseases via research, education, and advocacy (148).
